# Glycosylation Modulates Plasma Membrane Trafficking of CD24 in Breast Cancer Cells

**DOI:** 10.3390/ijms22158165

**Published:** 2021-07-29

**Authors:** Amanda Chantziou, Kostas Theodorakis, Hara Polioudaki, Eelco de Bree, Marilena Kampa, Dimitris Mavroudis, Elias Castanas, Panayiotis A. Theodoropoulos

**Affiliations:** 1 Laboratory of Biochemistry, School of Medicine, University of Crete, 70013 Heraklion, Greece; amanda_devet@hotmail.com (A.C.); polioudaki@uoc.gr (H.P.); 2 Institute of Molecular Biology & Biotechnology—FoRTH, Heraklion, 70013 Heraklion, Greece; kostas_theodorakis@imbb.forth.gr; 3 Department of Surgical Oncology, University General Hospital of Heraklion, 70013 Heraklion, Greece; debree@edu.uoc.gr; 4 Laboratory of Experimental Endocrinology, School of Medicine, University of Crete, 70013 Heraklion, Greece; kampam@uoc.gr (M.K.); castanas@uoc.gr (E.C.); 5 Department of Medical Oncology, University General Hospital of Heraklion, 70013 Heraklion, Greece; mavrudis@med.uoc.gr; 6 Laboratory of Translational Oncology, School of Medicine, University of Crete, 70013 Heraklion, Greece

**Keywords:** CD24, glycosylation, breast cancer, luminal and basal B cell lines, endocytic sorting

## Abstract

In breast cancer, expression of Cluster of Differentiation 24 (CD24), a small GPI-anchored glycoprotein at the cell periphery, is associated with metastasis and immune escape, while its absence is associated with tumor-initiating capacity. Since the mechanism of CD24 sorting is unknown, we investigated the role of glycosylation in the subcellular localization of CD24. Expression and localization of wild type N36- and/or N52-mutated CD24 were analyzed using immunofluorescence in luminal (MCF-7) and basal B (MDA-MB-231 and Hs578T) breast cancer cells lines, as well as HEK293T cells. Endogenous and exogenously expressed wild type and mutated CD24 were found localized at the plasma membrane and the cytoplasm, but not the nucleoplasm. The cell lines showed different kinetics for the sorting of CD24 through the secretory/endocytic pathway. N-glycosylation, especially at N52, and its processing in the Golgi were critical for the sorting and expression of CD24 at the plasma membrane of HEK293T and basal B type cells, but not of MCF-7 cells. In conclusion, our study highlights the contribution of N-glycosylation for the subcellular localization of CD24. Aberrant N-glycosylation at N52 of CD24 could account for the lack of CD24 expression at the cell surface of basal B breast cancer cells.

## 1. Introduction

Cluster of Differentiation 24 (CD24) is a small, heavily glycosylated glycophosphatidylinositol-anchored (GPI) protein. CD24 is considered a cancer stem cell marker [1] and is of critical importance in cancer, involved in cancer cell proliferation and metastasis, as well as immune suppression and escape [2]. 

CD24 primarily resides at the plasma membrane, and is highly expressed in various cancers, such as glioma, small cell lung carcinoma, urothelial carcinoma, ovarian and breast cancer [3]. Breast cancer-expressed CD24 was identified as a ligand for the adhesion receptor P-selectin on platelets and endothelial cells [4], through which it helps in the extravasation of tumor cells in circulation. CD24 modified with sialyl Lewis^x^ (sLe^x^) was shown to mediate P-selectin–dependent rolling in breast carcinoma in vitro and in vivo [4]. Furthermore, CD24 increases tumor cell proliferation, and it shows increased cell adhesion to fibronectin, collagens I and IV, and laminin through the activation of α3β1 and α4β1 integrin activity in breast cancer [5]. CD24 is involved in signaling pathways [6] through interaction with other signal transducer molecules that also reside in lipid rafts. Identified partners include P-selectin, L1, members of the Siglec family, TAG-1, members of the Src kinase family, and some DAMPs [3]. Through these interactions, CD24 mediates processes such as cancer development, invasion, and metastasis [6]. In gastric cancer cells, CD24 affects EGFR expression and localization by maintaining it in lipid rafts, thereby hindering its endocytosis and degradation; thus, it contributes to ERK activation, which enhances cell proliferation, survival, and motility [7]. Recently, CD24 was shown to promote immune evasion through its interaction with the inhibitory receptor Siglec-10 expressed by tumor-associated macrophages, revealing CD24 as a potent antiphagocytic (“don’t eat me”) signal in several cancers and demonstrating the therapeutic potential for CD24 blockade in cancer immunotherapy [8]. Cell populations expressing CD24 are identified as cancer stem cells (CSCs) in ovarian and colorectal cancer, nasopharyngeal carcinoma and pancreatic cancer cells [9,10,11,12]. In contrast, cells with low expression or absence of CD24 at the cell periphery were identified as CSCs in breast and prostate cancer [13,14] and in circulating tumor cells in the blood of breast cancer patients [15]. 

Mammary carcinoma is a heterogeneous neoplasm; multiple subtypes, displaying distinct morphologies and clinical attributes, were characterized, with the ductal carcinoma, originating from the mammary gland epithelium, being the most prevalent [16,17]. Clinically, ductal carcinoma is evaluated according to the expression profile of the estrogen (ER), progesterone (PR), and epidermal growth factor type 2 receptor (HER2), and it is subclassified into hormone-positive receptors (Luminal A, Luminal B), and HER2-positive and triple negative for hormone receptors (basal-like) [18]. The triple negative basal-like subtype represents approximately 10–15% of all mammary carcinomas, being characterized by high histological grade, high mitotic index, and low differentiation [19]. The triple negative subtype has a more aggressive clinical course compared with that of the other subtypes, and it is associated with a higher risk of distant metastasis recurrence and mortality [20]. 

Intracellular localization of CD24 was reported in tumor specimens of various cancers [21,22,23,24]. Cytoplasmic CD24 was shown to inhibit pancreatic cancer cell invasion by post-transcriptional regulation of BART through interaction with G3BP [24]. Additionally, CD24 was found in the nucleoplasm fraction of cancer cell lines from different tissues, including breast, lung, colon, and prostate cells, and it was suggested that the absence of CD24 from the cell surface could be attributed to its transport to the nucleus in human bladder cancer cell lines [25]. In prostate cancer, CD24 colocalizes and copurifies with NPM [21], a nucleolar phosphoprotein that shuttles between the cytoplasm and the nucleus during the cell cycle. This interaction destabilizes the ARF-NPM association, thereby causing tumor growth. Although these data argue for a nuclear localization of CD24, a mechanism responsible for its nucleocytoplasmic shuttling and a distinct nuclear function of CD24 were not elucidated yet. 

The premature CD24 molecule consists of 80 amino acids and contains two signal sequences: (i) an Endoplasmic Reticulum (ER)-signal sequence (1–26 aa), necessary for its ER translocation, and (ii) a GPI-anchor signal sequence (60–80 aa), which is essential for GPI-anchoring. After the signals’ removal, the remaining 33 residues constitute the mature peptide, which is heavily glycosylated. N-linked glycosylation of proteins starts in the ER lumen; the primary sequence of CD24 contains two sites consistent with the N-X-S/T motif (where X is not proline), which could possibly be N-glycosylated [26]. These sites are _36_NSS_38_ and _52_NAT_54_. The processing of N-glycans is also further resumed and completed in the Golgi apparatus, where additional O-linked glycosylation is initiated and completed [27]. GPI-anchoring is another CD24 post-translational modification event, which also starts in the ER, after recognition and cleavage of the GPI-anchor signal sequence by the multisubunit complex GPI transamidase and attachment of the protein on the GPI, whose maturation is conducted and finalized in the Golgi apparatus [28]. MALDI-TOF-MS analysis of CD24 showed an enrichment in the sialyl T-antigen O-glycan and six different N-glycans [29]. High-throughput analysis of the O-glycoproteome from kidney tissue samples identified threonine-41 as an O-glycosylation site, on which a disaccharide of the generic structure Hex-HexNAc was found [30]. A wide variety of glycosylation patterns of CD24 was identified among human and murine brains and brain cell lines [31].

A priori, N- and O-glycosylation patterns are highly variable among tissues [32], as well as subcellular compartments [33]. What remains obscure is the mechanism, which drives the subcellular localization of CD24 and particularly the role of its post-translational modifications. N-glycosylation and maturation of N-glycans are shown to be implicated in the localization of different integral [34,35,36,37,38,39] and GPI-anchored proteins [40,41,42,43] at the plasma membrane, whereas others remain unaffected [44].

In breast cancer the expression and localization of CD24 was related to the different subtypes [45] and the oncogenic properties [1] of breast cancer cells. Therefore, we aimed to investigate the role of N-glycosylation in the trafficking and subcellular localization of CD24 in luminal and basal B type of breast cancer cell lines. Using glycosylation inhibitors and point mutations, we found that CD24 expression at the plasma membrane is glycosylation- and cell type-dependent, whereas nuclear localization was completely absent.

## 2. Results

### 2.1. CD24 Localization in Breast Cancer Cells 

#### 2.1.1. Localization of Endogenous CD24 

**Figure 1:**
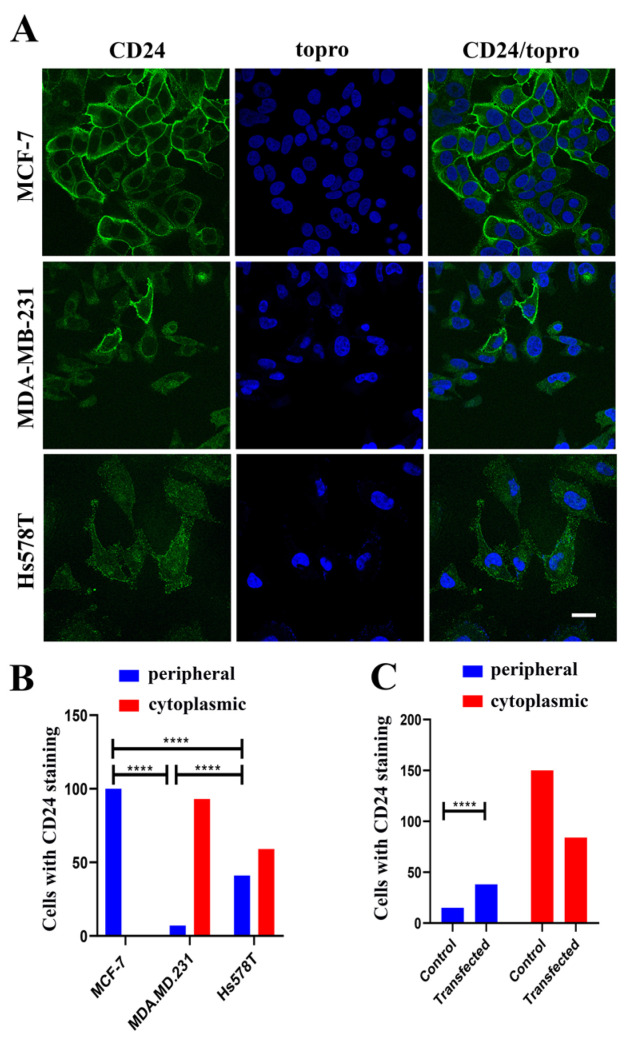
Localization of endogenous CD24 in breast cancer cell lines. (**A**) Representative cell staining for CD24, following immunofluorescence using the SN3 antibody. Scale bar, 20 μm. (**B**) Evaluation of peripheral staining of CD24 for each cell line. (**C**) Evaluation of peripheral staining of CD24 using SN3 antibody in MDA-MB-231 cells transfected or not with ER-tag CD24. Statistical significance (****) for *p* < 0.0001 with *t*-test.

Peripheral and cytoplasmic, but not nucleoplasmic, localization was observed among MCF-7, MDA-MB-231, and Hs578T breast cancer cell lines (as illustrated in Figure 1A), however, the cells with peripheral localization of CD24 were highly differentiated. All MCF-7 luminal-type cells expressed peripheral CD24; in contrast, only 40% of Hs578T cells and 7% of MDA-MB-231 basal, triple-negative, cells showed peripheral staining (as illustrated in Figure 1B). The low frequency of peripheral staining in MDA-MB-231 cells was not due to a technical artifact, since the percentage of cells showing peripheral staining was significantly increased in transfected cells, overexpressing CD24 (as illustrated in Figure 1C). Also, it seemed that CD24 was differently sorted among the cell lines, especially in MDA-MB-231 cells, in which CD24 localization was specific to a distinct intracellular compartment (as illustrated in Figure 1A).

#### 2.1.2. Localization of Transiently Expressed CD24

**Figure 2:**
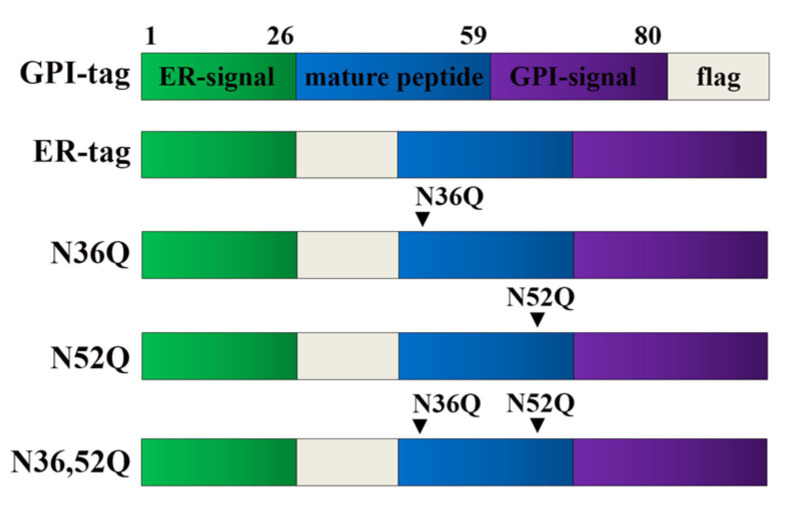
Schematic presentation of the different constructs used. For details on CD24 molecule and constructs, see text.

The localization of CD24 was investigated by transient transfection with two different plasmid constructs (as illustrated in Figure 2); one with the DDK-flag sequence inserted after the ER-signal sequence, able to produce the mature GPI-anchored CD24 (ER-tag CD24) and the other, not able to produce a GPI-anchor-modified CD24 due to the biochemical alteration of the GPI-anchor signal milieu by the presence of the acidic DDK-flag sequence inserted at the C-terminal of CD24 [46] (GPI-tag CD24). Localization of CD24 in the ER and Golgi compartments was estimated using specific markers, calnexin and GM130, respectively. Representative images showing localization patterns of CD24 following transfection with ER-tag CD24 are depicted in Figures S1 and S2. 

**Figure 3:**
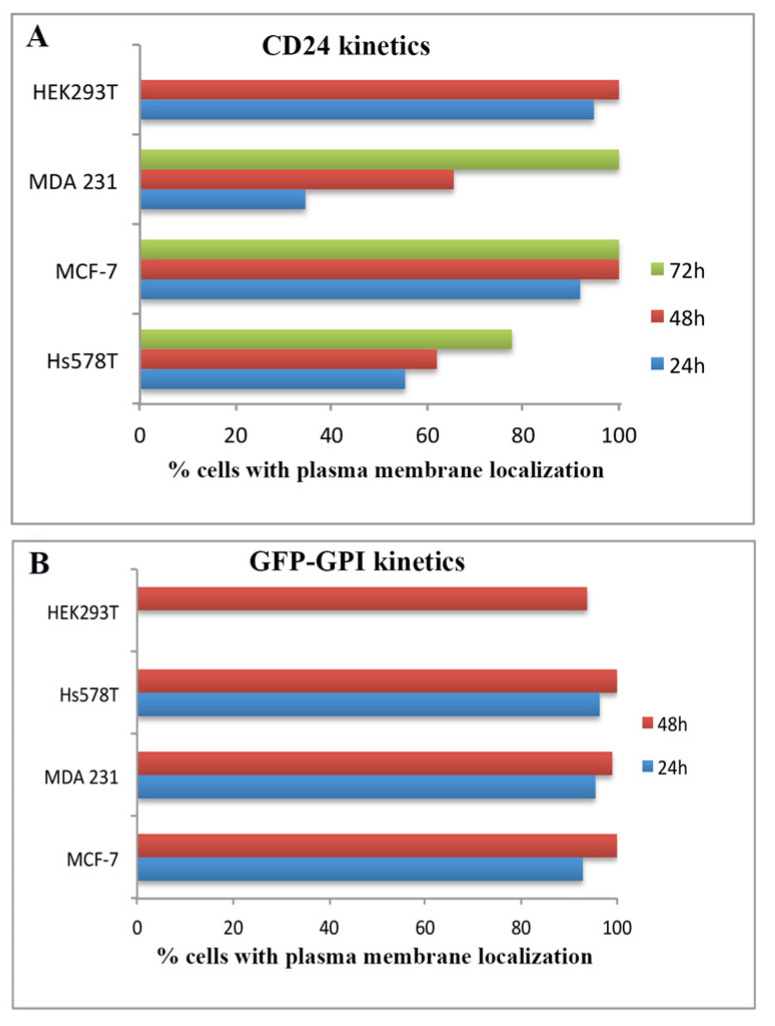
Kinetics of ER-tag CD24 (**A**) and GFP-GPI (**B**) in HEK293T and breast cancer cell lines. Diagrams show percentage of cells with plasma membrane localization independently of presence or not of ER-tag CD24 or GFP-GPI at cytoplasmic locations. Statistical analysis is presented in Table S1.

CD24 lacking a GPI-anchor showed predominantly cytoplasmic staining (as illustrated in Figure S3), while ER-tag CD24 was localized both at the cell periphery and in intracellular compartments stained with ER/vesicles and Golgi markers (as illustrated in Figures S1 and S2). Nuclear localization was not observed with any of the constructs used. Staining of either intact or membrane-permeabilized ER-tag transfected MCF-7 cells showed very similar percentages of complete, uniform, peripheral CD24 staining (79% for intact and 89% for permeabilized cells), indicating that the transfected CD24 is correctly localized at the extracellular side of the plasma membrane. Plasma membrane localized ER-tag CD24 was also recognized by the SN3 antibody (see Figure 1 and Figure S7), indicating that exogenously expressed CD24 bears similar epitope characteristics, especially sialylation. Time-dependent expression of ER-tag CD24 showed different kinetics for plasma membrane localization among the different breast cancer cell lines and HEK293T cells (as illustrated in Figure 3A, Table S1). Luminal type MCF-7 cells, as well as HEK293T cells, accumulate CD24 more rapidly at the cell membrane, as compared to that of basal type MDA-MB-231 and Hs578T cells. An explanation for such variations could be the glycosylation of CD24 and/or GPI-anchor processing, during the trafficking of CD24 from the ER to the plasma membrane. Plasma membrane localization kinetics of GFP-GPI, a protein whose primary sequence does not contain potential N-glycosylation sites, showed a rapid peripheral localization for all cell lines (as illustrated in Figure 3B, Table S1), indicating that GPI-anchoring and remodelling does not account for variable kinetics and a potential role of glycosylation of the protein core in CD24 trafficking through the secretory/endocytic pathway.

### 2.2. Effect of Glycosylation on CD24 Localization 

#### 2.2.1. N-Glycosylation Sites and Their Role for CD24 Localization

**Figure 4:**
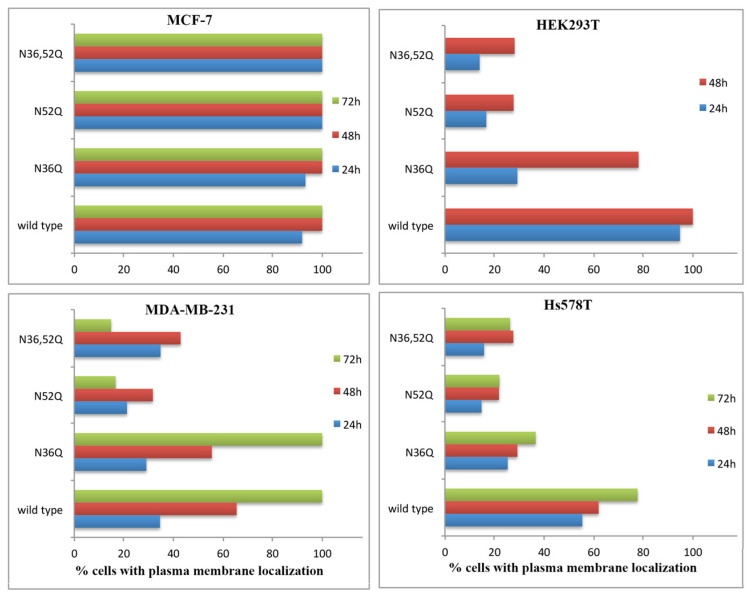
Plasma membrane localization kinetics of wild type, N36Q, N52Q, and N36,52Q CD24 in breast cancer cell lines and HEK293T cells. Diagrams show percentage of cells with plasma membrane localization independently of presence or not of ER-tag CD24 or CD24 mutants at cytoplasmic locations. Statistical analysis is presented in Table S2.

CD24 possesses two potential N-glycosylation sites, N36 and N52, among the 4 asparagine residues present in the 33 amino acid mature protein. The involvement of N-glycosylation in CD24 sorting was first investigated using single (N36Q, N52Q) and double (N36,52Q) point mutants of CD24 (as illustrated in Figure 4). 

Our data showed that N36 glycosylation is involved in CD24 sorting in Hs578T and HEK293T cells, but not in MCF-7 and MDA-MB-231 cells (as illustrated in Figure 4). At all timepoints, Hs578T cells transfected with N36Q-CD24 showed significantly lower plasma membrane localization (as illustrated in Figure 4, Table S2) and higher Golgi-localized CD24 compared to that of wild type CD24 transfected controls. Similar results were obtained in HEK293T cells, a cell line negative for CD24, transfected with N36Q-CD24 (as illustrated in Figure 4, Table S2). 

Asparagine-to-glutamine mutation at N52 showed a more dramatic effect on CD24 localization; MDA-MB-231, Hs578T and HEK293T cells showed a significant decrease of CD24 plasma membrane localization. A similar pattern in the three cell lines was observed by the double N36,52Q CD24 mutation, confirming the significance of the N52 glycosylation for the localization of the CD24 (as illustrated in Figure 4, Table S2). Plasma membrane localization was restored following incubation of N52Q transfected HEK293T cells with the proteasome inhibitor MG132 (as illustrated in Figure S4), suggesting a reduced stability at the plasma membrane of mutated N52Q compared to that of wild type CD24. MCF-7 cells did not show any phenotypic variation upon transfection with wild type, N36Q, N52Q, or N36,52Q CD24. 

In sum, these data argue for the absence of involvement of N-glycosylation in the sorting of CD24 in MCF-7 cells and for an important role of N52 glycosylation in the sorting and stability of CD24 in basal B cell lines and HEK1293T cells. N-glycosylation had no effect on the nuclear localization of CD24 under the studied conditions. 

#### 2.2.2. The Impact of N-Glycan Maturation and O-Glycosylation on CD24 Localization 

##### Effect of Kifunensine 

The impact of N-glycan maturation in CD24 sorting was investigated using kifunensine, a mannosidase inhibitor involved in N-glycosylation processing. 

**Figure 5:**
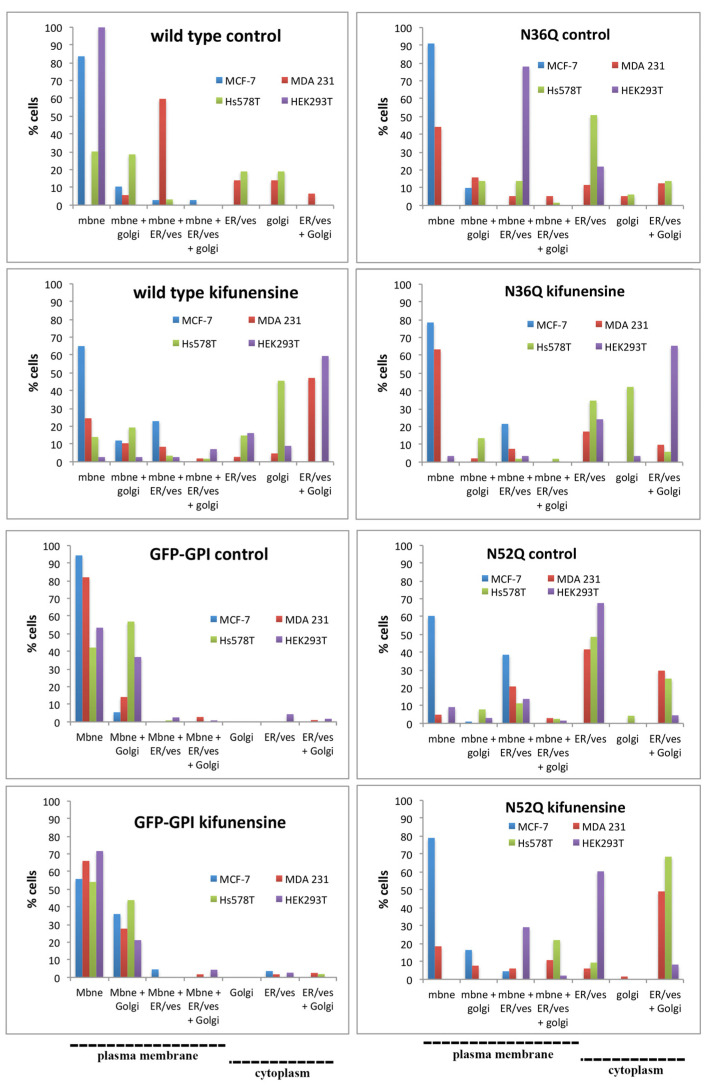
Effect of kifunensine on the subcellular localization of CD24. Cells transfected for 48 h with wild type, N36Q or N52Q CD24 in presence or absence of kifunensine. Then, cells were immunolabeled with anti-flag and anti-GM130 antibodies and subcellular localization of CD24 was analyzed using confocal microscopy. As a control, cells were also transfected with GFP-GPI and subcellular localization was assessed with anti-GFP and anti-GM130 immunolabelling. Seven distinct cell phenotypes were analyzed for each cell line/construct/treatment as follows: (1) cells with only plasma membrane staining (mbne); (2) cells with plasma membrane plus golgi staining (mbne + golgi); (3) cells with plasma membrane plus ER or vesicular staining (mbne + ER/ves); (4) cells with plasma membrane plus ER or vesicular plus golgi staining (mbne + ER/ves + golgi); (5) cells with only ER or vesicular staining (ER/ves); (6) cells with only golgi staining (golgi); (7) cells with ER or vesicular plus golgi staining (ER/ves + golgi). Sum of cells with phenotypes 1, 2, 3 and 4, or 5, 6 and 7 constitute group of cells with plasma membrane or cytoplasmic CD24, respectively. Statistical analysis of different phenotypes is presented in Figure S5 and Table S3.

MCF-7 cells transfected with wild type, N36Q- or N52Q-CD24 did not show significant changes in the percentages of cells with plasma membrane or cytoplasmic localization, although an increase of cells expressing both cytoplasmic and peripheral wild type (*p* < 0.01) and N36Q-CD24 (*p* < 0.05) was observed following kifunensine treatment (as illustrated in Figure 5 and Figure S5A,B). In contrast, sorting and localization of CD24 at the plasma membrane was affected by kifunensine in all other studied cell lines. 

Kifunensine changed almost completely the localization, from the plasma membrane to the cytoplasm, in both wild type (*p* < 0.0001) and N36Q-CD24 (*p* < 0.0001) transfected HEK293T cells, whereas it had not any significant effect on the membrane or Golgi localization of N52Q-CD24 transfected cells (as illustrated in Figure 5 and Figure S5A). These data highlight the significance of both the presence and maturation of glycans attached to N52, but not to N36, for the sorting of CD24 to the plasma membrane in HEK293T cells. 

Compared to that of nontreated cells, wild type and N36Q-CD24 transfected Hs578T cells treated with kifunensine showed decreased (although no statistically significant for N36Q-CD24) percentages (38% and 41%, respectively) of cells with plasma membrane localization of CD24 (as illustrated in Figure 5 and Figure S5A) and considerably increased number of cells (1.5 and 7 times more, respectively) with an exclusively Golgi localization (as illustrated in Table S3). In contrast, no changes in the percentages of cells with plasma membrane localization were found in N52Q-CD24 transfected cells treated (22%) or not (21.8%) after kifunensine treatment. However, a statistically significant increase in cells with Golgi plus ER and/or Golgi CD24 was measured following kifunensine treatment in cells transfected with either N36Q-CD24 or N52Q-CD24 (as illustrated in Table S3). These data indicate a maturation process in both N36 and N52 glycans; however, glycans attached to N52 (in cells transfected with N36Q-CD24) might have an additional role for CD24 plasma membrane localization. 

MDA-MB-231 cells treated with kifunensine showed statistically significant (*p* < 0.01) decreased number of cells (31%) with CD24 plasma membrane localization when transfected with WT-CD24 (as illustrated in Figure 5 and Figure S5A). No significant changes in plasma membrane localization were observed in cells transfected with either the N36Q-CD24 or N52Q-CD24 plasmids, although a significant increase in cells with both Golgi plus ER and/or exclusively Golgi localization was measured following kifunensine treatment (as illustrated in Table S3). These data are probably indicative of a maturation process of the N-glycan attached in both N36 and N52 residues of CD24. 

Treatment of cell lines with kifunensine did not change the ratio of cells presenting either plasma membrane or exclusively cytoplasmic localization of GFP-GPI (as illustrated in Figure 5 and Figure S5A). However, kifunensine affected the ratio of cells with exclusively plasma membrane or with both plasma membrane and cytoplasmic GFP-GPI localization, either promoting (in HEK293T cells, *p* < 0.05) or retarding (in MCF-7, *p* < 0.0001 and MDA-MB-231 cells, *p* < 0.05) the GFP-GPI localization at the plasma membrane (as illustrated in Figure 5 and Figure S5B), suggesting an effect on N-glycosylation of enzymes involved in GPI maturation or other sorting machinery. 

All in all, these findings show the involvement of N-glycan processing in CD24 for the cell lines subjected to N-glycosylation, but not for MCF-7 cells where the effect of kifunensine was minimal and very similar to those observed for the nonglycosylated GFP-GPI. 

##### Effect of Benzyl-α-GalNAc

**Figure 6:**
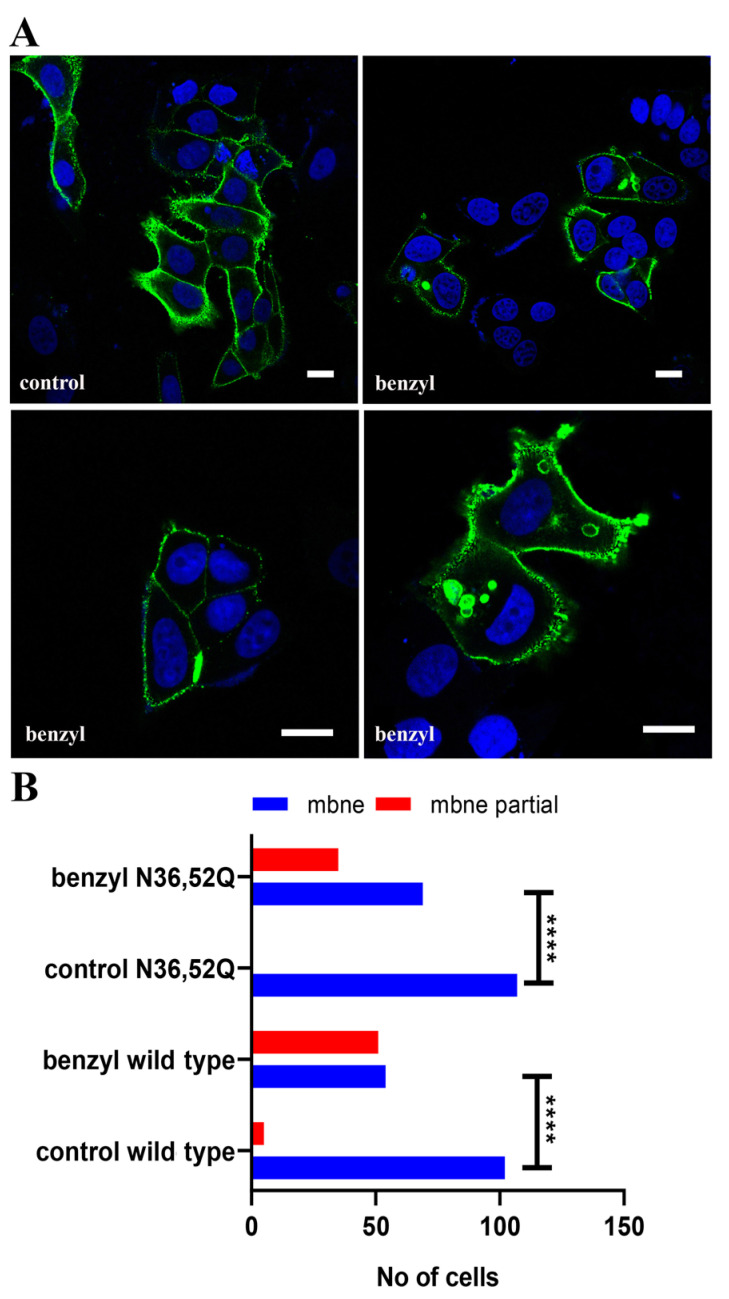
Effect of benzyl-α-GalNAc on subcellular localization of CD24 in MCF-7 cells. (**A**) Representative combined images of cells stained with anti-flag and topro showing localization of wild type (ER-tag) CD24 in transfected control (control) and cells treated with 5 mM benzyl-α-GalNAc for 48 h (benzyl). Scale bars, 10 μm. (**B**) Diagram presenting absolute number of cells with complete (mbne) or partial (mbne partial) localization of CD24 following transfection with wild type (ER-tag) and double N36, 52Q mutant in absence (control) and presence of 5 mM benzyl-α-GalNAc for 72 h. Statistical significance (****) for *p* < 0.0001 with *t*-test.

The contribution of CD24 O-glycosylation and/or N-glycan sialylation for the efficient sorting and targeting of the protein at the plasma membrane was investigated using benzyl-α-GalNAc [47]. MCF-7 cells transfected with wild type CD24 and treated with benzyl-α-GalNAc for 24 h showed a 30% increase of cells with, in addition to plasma membrane, ER/Golgi-localized CD24. After 48 h of treatment, 25% of cells showed vesicular intracellular accumulation of CD24 with or without dense accumulation or large deficits of CD24 at the plasma membrane (as illustrated in Figure 6). At 72 h of treatment, about half of cells showed a nonuniform distribution of CD24 at the plasma membrane, with areas with significantly reduced CD24 mostly located at adjacent cells’ contact sites. Very similar results were obtained following transfection with N36,52Q-CD24, where 35% of cells showed this partial localization at the plasma membrane (as illustrated in Figure 6). These data indicate that changes in the topology of CD24 are most probably irrelevant to N-glycan modification but possibly related to the effect of benzyl-α-GalNAc on CD24 O-glycosylation.

MDA-MB-231 and Hs578T cells treated with benzyl-α-GalNAc for 48 h did not show the phenotypic variations observed in MCF-7 cells. However, compared to that of untreated cells, increased percentages of cells with membrane localization (14% and 25% for Hs578T and MDA-MB-231 cells, respectively) and similar decreased percentages of cells with exclusively cytoplasmic CD24 (12% and 24% for Hs578T and MDA-MB-231 cells, respectively) were found (as illustrated in Figure S6). Although not effective in altering plasma membrane localization, benzyl-α-GalNAc was shown to modify CD24 sialylation. Double staining of cells with flag and SN3 antibodies showed cells stained at the plasma membrane with anti-flag but not with SN3 antibody (as illustrated in Figure S7). 

## 3. Discussion

Differential expression and subcellular localization of CD24 in cancer cells is implicated in tumor initiation, cancer cell proliferation and metastasis, and immune suppression and escape. Our work reveals that glycosylation modulates the localization of CD24 in breast cancer cell lines. We found that N-glycosylation was critical for CD24 expression and localization at the cell surface of basal B cell lines since loss of glycosylation at asparagine-52 resulted in the lack of plasma membrane CD24. In contrast, plasma membrane localization of CD24 in the luminal type MCF-7 cells was independent of N-glycosylation. Luminal and basal B cell lines exhibit differential expression of glycoproteins [48], hypoxic conditions affect N-glycosylation, namely glycosyltransferases expression and increase in sialylation and branching of glycans [49], and glycoproteome alterations in breast cancer have prognostic value [50]. The fact that CD24 was robustly localized at the plasma membrane in MCF-7 cells and no condition was able to abolish this phenotype indicates that CD24 trafficking in these cells is directed by mechanisms independent of N-glycosylation, and may, in fact, rely on protein-protein or protein-GPI interactions, which emerge as early as the protein is found in the ER. In contrast, in basal B and HEK293T cells, CD24 localization appears to have an additional layer of trafficking regulation by virtue of N-glycosylation. This differential effect could be attributed to differential expression levels of glycosyltranferases, hypoxic conditions, EMT status, and other protein interactors to name a few. One could only speculate about the functionality of the variety of mutated/post-treatment species that reach the cell surface of MCF-7 cells, and, in parallel, of those species intracellularly retained in the rest of the cell lines. Furthermore, what becomes evident is the importance of the N52 residue, in CD24 trafficking. Given the proximity of the residue to the GPI-anchor signal sequence, this site may be involved in the correct GPI-anchoring and folding of the molecule.

The subcellular localization of CD24 was studied in a variety of cancer types and one could be at liberty to argue that, overall, CD24 was found to reside at the plasma membrane, the cytoplasm, and the nucleus. These compartments were linked to distinct functions, namely, involvement in specific signal transduction pathways. What is evidenced in our study is that endogenous and transfected CD24 can localize at the plasma membrane.

Nuclear CD24 was described in breast tumor biopsies [51] and the existence of nuclear CD24 species could explain the aggressiveness of bladder cancer cells, negative for surface CD24 [25]. However, nuclear localization studies can be precarious, since technical artifacts can emerge [52], and also, nuclei isolation experiments with CD24 are hardly accompanied by purity verification using ER markers. In this study, we found no indication of a nuclear localization, under no conditions with any given construct, except for an emphasis in immunofluorescence experiments at the ER/nuclear envelope compartment, under some conditions. Localization at the nucleoplasm was definitively absent.

What is more provocative is the cytoplasmic localization of CD24. As with localization in the nucleus, there are studies reporting cytoplasmic localization in tumors and cancer cell lines. Again, this could be a technical artifact if e.g., the ER membranes are harshly disrupted by detergents; however, CD24 localized at the cytosol can be found in stress granules [24]. Cytosol-localizing CD24 is intriguing in terms of how it “escaped” ER-translocation, how it is post-translationally modified, and what are its interactors and functionality. In this study, CD24 was primarily found to localize at membranous compartments, be that the plasma membrane, Golgi, vesicles, or ER/nuclear envelope. Exception to this is the GPI-tag CD24, which most likely resides at the ER lumen and is potentially secreted due to the acidic DDK tag altering the hydrophobic nature of the GPI-anchor signal sequence and hindering the GPI-anchoring [46]. This persistence in membranous localization shows that the exogenous protein is, in fact, behaving canonically. What further supports this argument is the staining of exogenously expressed CD24 with SN3 antibody, which reflects that sialic acid residues are present, and therefore the molecule is probably functional.

For these reasons put forth, we used an immunofluorescence protocol that relies on two-step fixation and staining, and a very brief permeabilization step with methanol between them [53] that produced consistent phenotypes. Methanol was chosen among detergents such as saponin and digitonin as the most satisfactory in terms of reproducibility for plasma membrane and intracellular organelles’ integrity and staining (not shown).

Maturation of N-glycans was shown to be important for plasma membrane proteins localization and function. Endocytosis or surface retention of EGFR and cytokine receptors were reported to be regulated by N-glycan processing [54,55]. The important role of mannosidase in N-glycan processing was shown for the trafficking of sodium iodide symporter to the plasma membrane in breast cancer cells, since inadequate mannose processing by mannosidase impaired its trafficking through the endocytic pathway, its localization at the cell membrane, and its functionality [34]. The fact that mannosidase activity is a prerequisite for formation of complex type N-glycans, which comprise sugars necessary for the function of peripheral CD24, could explain the observation that CD24 had no prognostic influence in tumors with low mannosidase expression in breast cancer patients [56]. Our data indicate that depending on the cell line, either the presence and/or the maturation of specific N-glycans are implicated in the sorting of CD24 to the plasma membrane. In MCF-7 cells, the typical localization of CD24 at the cell periphery is not modified by the presence of N-glycans or the inhibition of their maturation to hybrid or complex type at both glycosylation sites.

Benzyl-α-GalNAc was reported to affect sialylation during maturation of N-glycans, in addition to its primary function in O-glycosylation [47]. The very similar data we obtained for wild type and N36,52Q CD24 in MCF-7 cells, indicate that benzyl-α-GalNAc most probably affects only the process of O-glycosylation. The phenotype observed following benzyl-α-GalNAc treatment in MCF-7 cells, namely the severely unequal distribution of CD24 at plasma membrane, could be the result of altered O-glycosylation of CD24 and interacting proteins or alternative post-translational modifications that take place in lieu of O-glycosylation, which hinder proper interactions that allow for CD24 uniform lateral diffusion. In MDA-MB-231 and Hs578T cells, the absence of plasma membrane alterations following benzyl-α-GalNAc treatment indicate probably different O-glycosylation requirements for CD24, or other interacting proteins implicated in the organization of CD24 at the plasma membrane of basal B breast cancer cells.

Our study shows, for the first time, the importance of N-glycosylation for the localization of CD24 at the cell periphery of breast cancer cells. Also, it highlights the differential contribution of the two sites of N-glycosylation in CD24 sorting and the different requirements among the different breast cancer cell lines. We found that absence of glycosylation at N52 results in loss of peripheral CD24 in MDA-MB-231 and Hs578T breast cancer cells, as well as in HEK293T cells, reminiscent of what we observe for endogenous CD24 in these cells. Although we cannot propose a mechanism, an explanation for this effect could be folding defects due to the glycosylation alone or in combination to modifications in other residues, such as phosphorylation. Particularly, it appears that N52 is a key residue in directing CD24 localization, and it should be taken into account when considering the effects of other post-translational modifications. The dynamic nature of phosphorylation is a very promising field for investigating the role of post-translational modifications in the localization and stability of CD24. So far, no residue of CD24 was identified as phosphorylated, although its primary sequence harbors potential phosphorylation motifs. All in all, the field of post-translational modifications of CD24 in directing the subcellular localization of CD24 is a very intriguing, but also perplexing task, which is under investigation in our laboratory.

## 4. Materials and Methods

### 4.1. Cell Cultures and Treatments 

HEK293T cells and breast cancer cell lines of luminal (MCF-7) or basal B (MDA-MB-231 and Hs578T) subtype used in this study were procured from American Type Tissue Culture Collection (Manassas, VA, USA). MDA-MB-231, Hs578T, and HEK293T cells were cultured in Dulbecco’s modified Eagle’s medium (DMEM) medium and MCF-7 in DMEM plus 10 g/mL insulin at 37 °C in a humidified atmosphere containing 5% CO_2_. Culture media were purchased from Gibco (Dublin, Ireland) and were supplemented with 10% heat-inactivated fetal bovine serum and 1% penicillin/streptomycin.

Cells were treated with kifunensine (10009437, Cayman Chemical, MI, USA) 10 μΜ for 48 h and benzyl-α-GalNAc (B4894, Sigma–Aldrich, Taufkirchen, Germany) 5 mM for 48 h or 72 h. MG132 (10012628, Cayman Chemical, Ann Arbor, MI, USA) was added at 1 μM for 24 h. 

### 4.2. CD24 Constructs and Transfection

CD24 (NM 013230) human tagged ORF clone was supplied by Origene (RC209542, Rockville, MD, USA). In this construct, termed in the text as GPI-tag CD24, DDK-flag sequence was inserted in the C-terminal of the molecule after the GPI-anchor sequence. Therefore, we constructed a plasmid, designated in the text as ER-tag, with DDK-flag sequence inserted in the N-terminal of the mature CD24 after the ER signal sequence. Point mutagenesis at N36 and N52 to Q was performed using the approach as previously described [57] individually in the ER-tag CD24 expression construct using specific forward and reverse primers; for N36Q (forward) 5′-CTTCAAGTCAGTCCTCCCAGAGTACTTCCAACTCTGGGTTG-3′ (reverse) 5′-GGGAGGACTGACTTGAAGTTCCAGTTGTTGTTTCACTTCCG-3′ and for N52Q 5′-ATCCAACTCAGGCCACCACCAAGGCGGCTGGTGGTGCCCTG-3′ and 5′-TGGTGGCCTGAGTTGGATTTGGGGCCAACCCAGAGTTGGAAGTAC-3′, forward and reverse, respectively. For the construction of the double mutant N36,52Q, we used the single mutant plasmids and the corresponding set of primers. The PCR products were transformed into DH10b competent cells using electroporation. Plasmids were extracted and DNA sequence of wild type and mutated constructs were confirmed by DNA sequencing using specific primers; VP1.5 (Forward 5′-GGACTTTCCAAAATGTCG-3′), XL39 (5′-ATTAGGACAAGGCTGGTGGG-3′ Reverse) CMV (Forward CGCAAATGGGCGGTAGGCGTG). All CD24 constructs used in this study are schematically presented in Figure 2. The GFP-GPI construct was a gift of Prof C. Zurzolo [58]. 

For transient transfection, plasmid DNA was delivered to cells using the nonliposomal lipid reagent, Attractene (301005, Qiagen, Germantown, MD, USA). Transfections were performed according to the manufacturers’ instructions. In brief, cells were seeded on coverslips in 24 well plates. When cultures reached approximately 80% confluency, they received 0.4 μg DNA and 1.5 μL of Attractene, per well. After a 6 h incubation the transfection medium was replaced by fresh medium. Cell cultures were used 24 to 72 h post-transfection for immunofluorescence analysis. 

Treatment of transfected cultures with inhibitors, diluted in the cells’ media, was performed during the transfection and after the renewal of the transfection medium until the indicated time point. 

### 4.3. Immunofluorescence

To preserve and detect both membranous and intracellular localization of CD24, cells were labeled with primary and secondary antibodies twice; first for the peripheral, extracellular CD24 and secondly for the intracellular CD24, after postfixation and very rapid permeabilization with methanol. Detergents were omitted from all buffers during the procedure to avoid CD24 mobilization and abnormal localization [52]. In brief, cells grown on coverslips were rinsed with PBS, fixed with 1% methanol-free formaldehyde for 20 min and subsequently blocked with blocking buffer (PBS, pH 7.4, MgCl_2_ 2 mM and fish skin gelatin 0.5%) for 10 min. Then, cells were incubated with primary antibodies for 45 min, rinsed with PBS and then secondary antibodies for 45 min. Cells were fixed, for a second time, with 1% methanol-free formaldehyde for 20 min and permeabilized instantaneously with ice-cold pure methanol followed by two washes with PBS. Afterwards, the blocking and staining incubations were repeated as described above. Finally, cells were counterstained with topro. Primary antibodies included anti-CD24 (SN3 clone, sc-19585, Santa Cruz Biotechnology, Heidelberg, Germany) which recognizes a glycan, probably sialic acid epitope on CD24 [59], anti-flag (M2 clone, F3165, Sigma–Aldrich, Taufkirchen, Germany), anti-flag (rabbit, D-8, sc-807 Santa Cruz Biotechnology, Heidelberg, Germany), anti-GM130 (rabbit polyclonal, NBP2-53420, Novus Biologicals, Centennial, CO, USA) and anti-calnexin (rabbit polyclonal, NB100.1974, Novus Biologicals, CO, USA). 

Slides were examined under a conventional epifluorescence microscope (Leica) using a 40× objective lens with oil immersion and were further analyzed by confocal (Leica SP2) microscopy. To prevent any signal interference (green, red and blue) generated by the different emission spectra, the detection of each one of the markers was performed by sequential laser confocal scan. Phenotypic analysis consisted in the evaluation in duplicate of the subcellular localization of endogenous and exogenously expressed CD24, mainly at the plasma membrane, ER and Golgi compartments, and nucleus in at least 50 cells. 

### 4.4. Statistical Analysis

Data were analysed with GraphPad Prism version 8.0.1 (GraphPad Software Inc., San Diego, CA, USA). *p* < 0.05 was taken as the statistical threshold.

## Supplementary Materials

The Supplementary Materials are available online at https://www.mdpi.com/article/10.3390/ijms22158165/s1.
