# Supplementary material for: Glycosylation Modulates Plasma Membrane Trafficking of CD24 in Breast Cancer Cells
Source: Int J Mol Sci. 2021 Jul 29;22(15):8165. doi: 10.3390/ijms22158165 (PMC8347636; doi:10.3390/ijms22158165)
Supplement: Supplementary file 1 [file ijms-22-08165-s001.zip › ijms-1313733-supplementary.pdf]

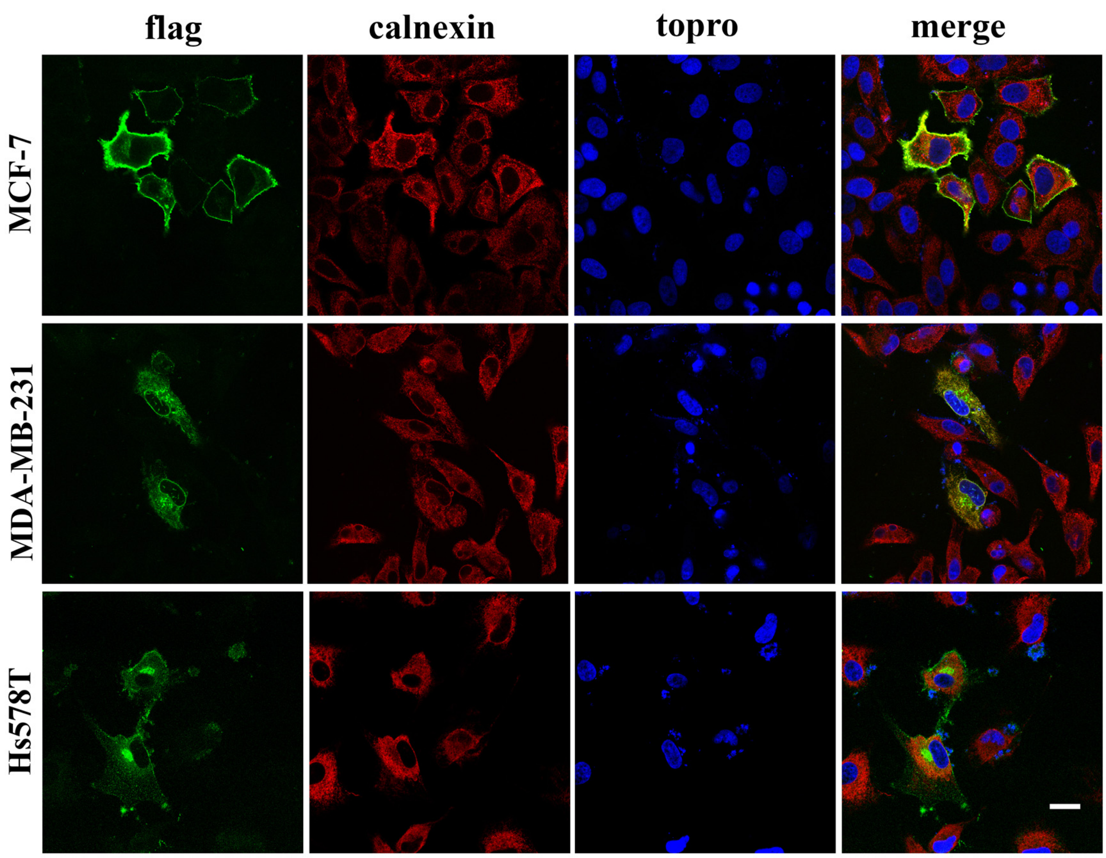

Figure S1. *Staining of breast cancer cells with the ER marker calnexin.* Representative images of Hs578T, MCF-7 and MDA-MB-231 cells transfected with ER-tag CD24 and stained with anti-flag, anti-calnexin antibodies and topro, are shown. In MCF-7 row, cells with plasma membrane or plasma membrane plus ER/vesicles are shown. In MDA-MB-231 row, both cells show ER/vesicles pattern. In Hs578T row both cells show plasma membrane plus ER/vesicles pattern. Scale bar, 20 μm.

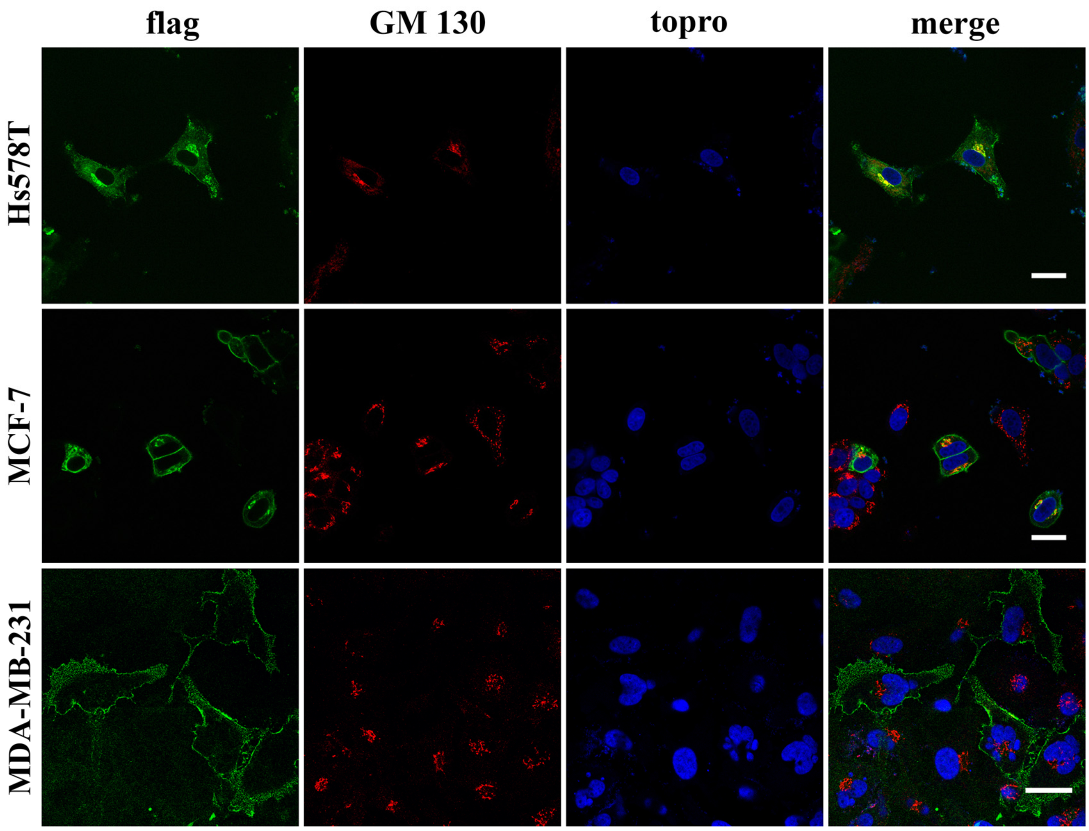

Figure S2. *Staining of breast cancer cells with the Golgi marker GM130.* Representative images of Hs578T, MCF-7 and MDA-MB-231 cells transfected with ER-tag CD24 and stained with anti-flag, anti-GM130 antibodies and topro, are shown. In Hs578T row, cells show plasma membrane plus Golgi pattern. In MCF-7 row, cells with plasma membrane or plasma membrane plus Golgi are shown. In MDA-MB-231 row, cells show plasma membrane pattern. Scale bars, 20µm.

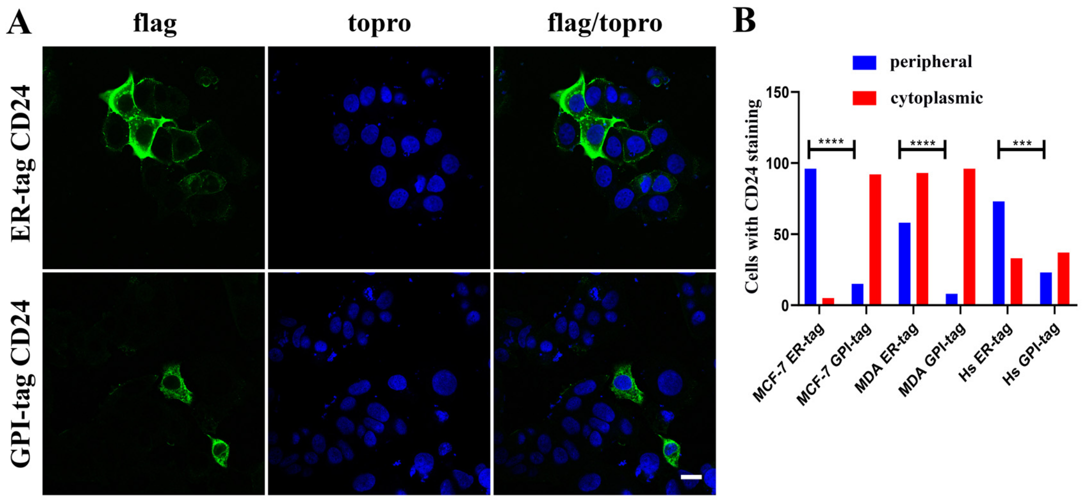

Figure S3. *Localization of ER-tag and GPI-tag CD24 in breast cancer cell lines.* (A) Staining of ER-tag and GPI-tag CD24 transfected MCF-7 cells with anti-flag antibody. In the ER-tag panel all cells show peripheral staining, while in the GPI-tag panel cells with cytoplasmic staining are shown. Scale bar, 15μm. (B) Evaluation of the peripheral staining for each cell line transfected with ER-tag or GPI-tag CD24 for 24 hours. Statistical significance (****) for p<0.0001 and (***) for p<0.001 with t-test.

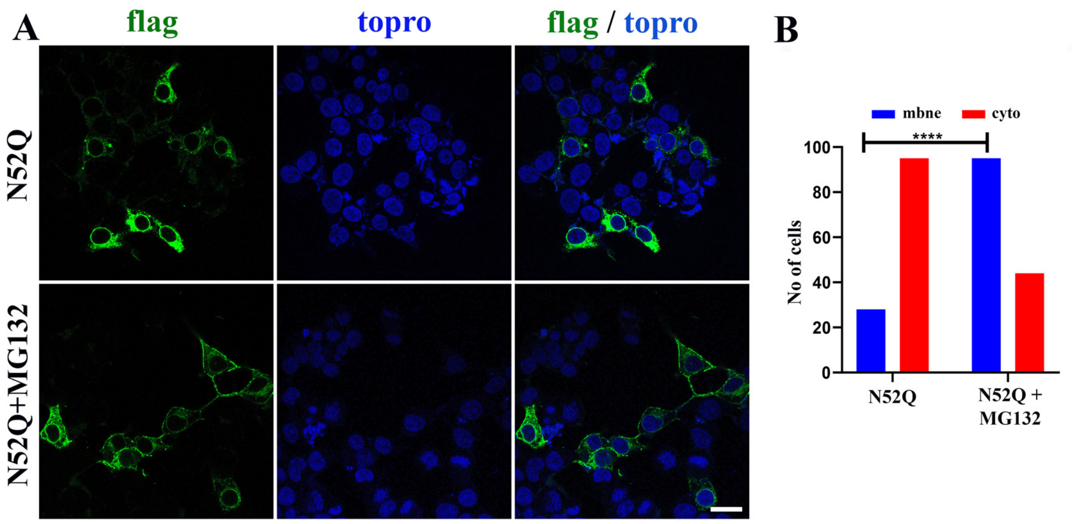

Figure S4. *Reversal of N52Q loss of plasma membrane localization by MG132.* (A) Representative images of HEK293T cells transfected with N52Q CD24 for 24h in the presence or absence of 1μM MG132. Scale bar, 20μm. (B) Absolute number of cells with plasma membrane or cytoplasmic staining, following transfection with N52Q CD24 for 24h in the presence or absence of 1μM MG132. The difference in membrane staining between N52Q and N52Q+MG132 is statistically significant (p<0.0001 with t-test).

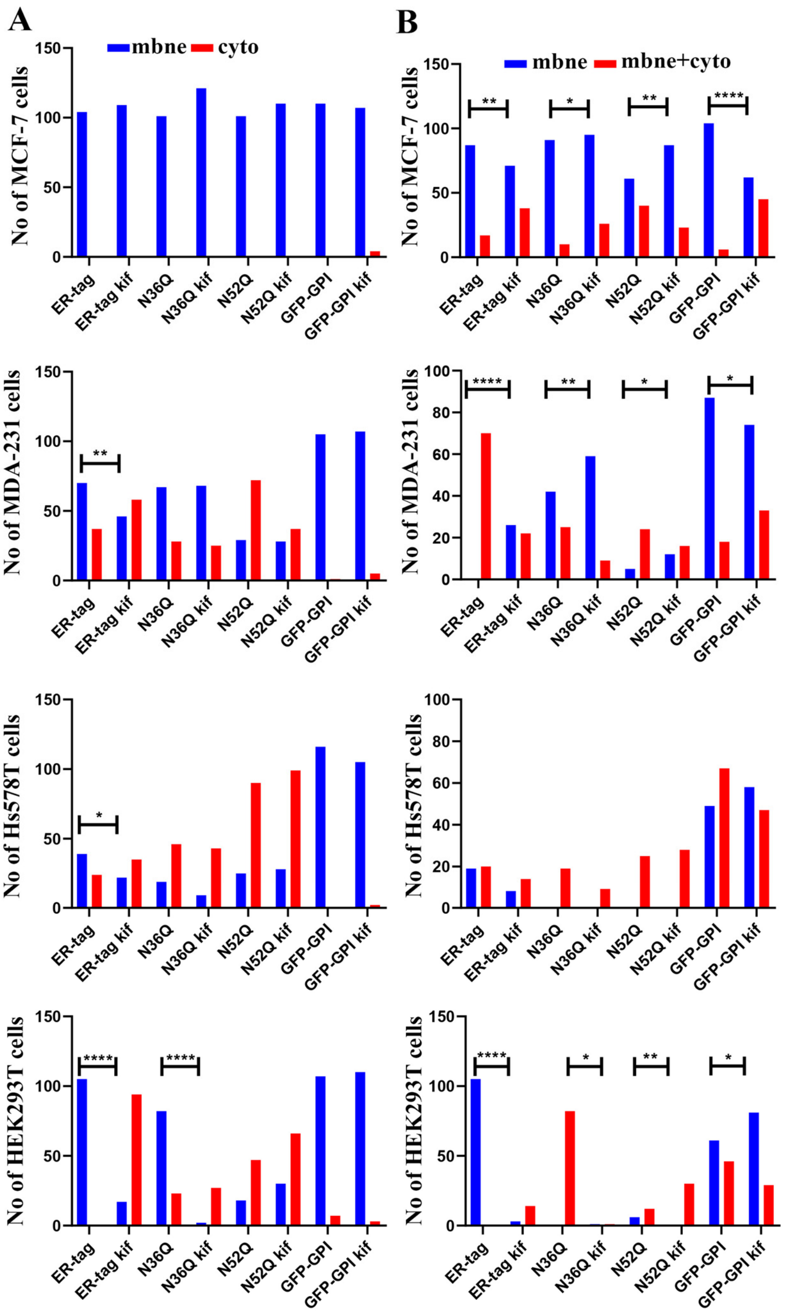

Figure S5. *Statistical analysis of data shown in Figure 5.* MCF-7, MDA-MB-231, Hs578T and HEK293T cells transfected for 48h with wild type, N36Q, N52Q CD24 and GFP-GPI in the absence or presence of kifunensine (kif) were immunolabeled with anti-flag and anti-GM130 and the plasma membrane or cytoplasmic localization was analyzed using confocal microscopy. (A) Analysis of cells with cytoplasmic (cyto) and cells with plasma membrane staining independently of the presence or not of cytoplasmic staining (mbne). The latter phenotype (mbne) comprises cells with plasma membrane but not cytoplasmic staining and cells with plasma membrane with any other cytoplasmic pattern. (B) Analysis of cells with plasma membrane but not cytoplasmic staining (mbne) and cells with plasma membrane with any other cytoplasmic pattern (mbne+cyto). Statistical significance (****) for $p<0.0001$, (**) for $p<0.01$ and (*) for $p<0.05$ with t-test.

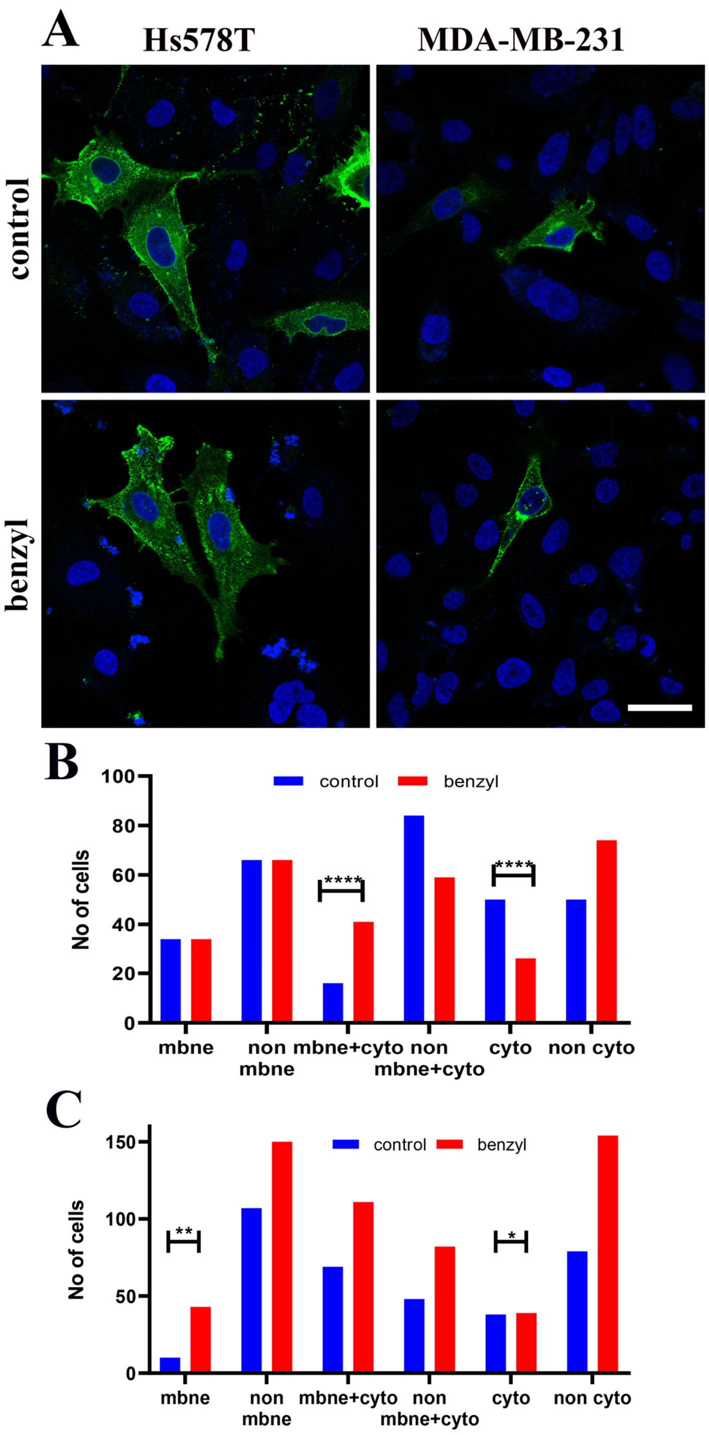

Figure S6. *Effect of benzyl-α-GalNAc on the subcellular localization of CD24 in MDA-MB-213 and Hs578T cells transfected with wild type CD24*. (A) Representative images of cells stained with anti-flag antibody and topro. Scale bar, 20μm. (B, C) Absolute number of MDA-MB-231 (B) and Hs578T (C) cells with plasma membrane (mbne), both plasma membrane and cytoplasmic (mbne+cyto) and only cytoplasmic (cyto) localization of flag-CD24 in DMSO (control) and 5mM benzyl-α-GalNAc (benzyl) treated for 48h cells. Statistical significance (****) for p<0.0001, (**) for p<0.01 and (*) for p< 0.05 with t-test.

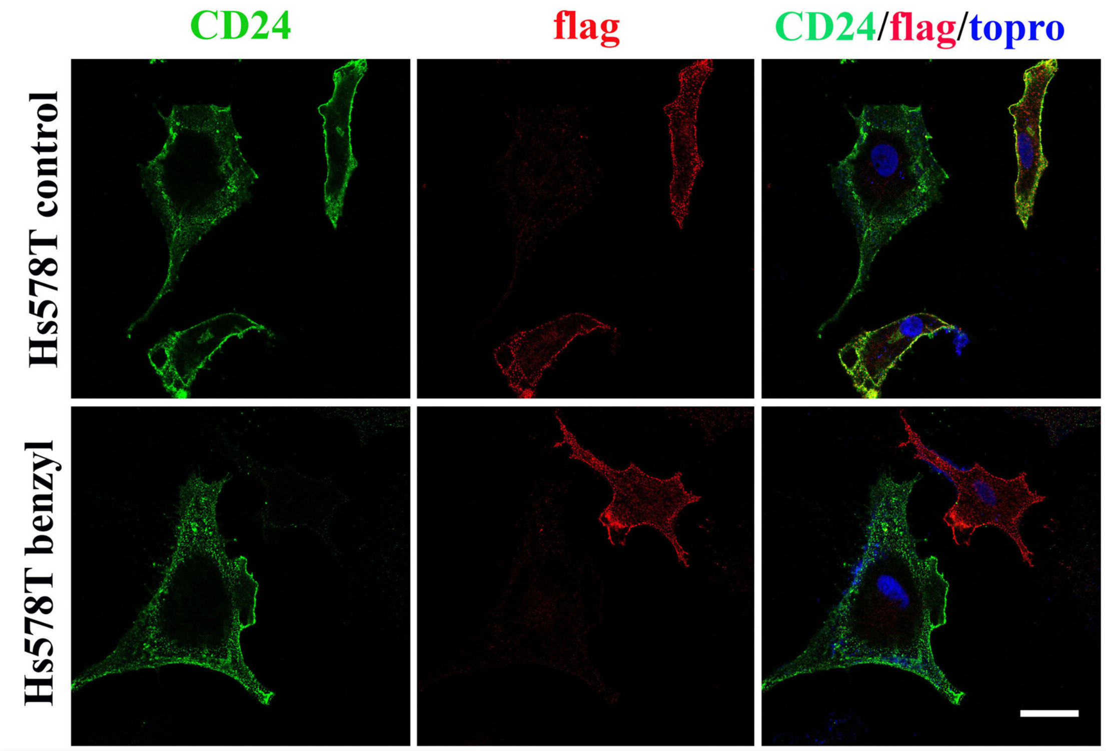

Figure S7. *Effect of benzyl-α-GalNAc on the subcellular localization of endogenous and transfected CD24 in Hs578T cells.* Cells transfected with ER-tag CD24 and treated with 5 mM benzyl-α-GalNAc for 48 hours were stained with both mouse SN3 (CD24) and rabbit anti-flag antibodies. Representative images show that following benzyl-α-GalNAc, plasma membrane localized ER-tag CD24 is not recognized by SN3 antibody. Scale bar, 20µm.

Table S1. Statistical analysis (t-test) of the data presented in Figure 3.

		24h	48h	72h
**MCF7 vs MDA**	GPI-GFP	ns	ns	
	ER-tag	****	****	ns
**MCF7 vs Hs**	GPI-GFP	ns	ns	
	ER-tag	****	****	****
**MCF7 vs HEK**	GPI-GFP		ns	
	ER-tag	ns	ns	
**MDA vs HEK**	GPI-GFP	ns	ns	
	ER-tag	****	****	
**MDA vs Hs**	GPI-GFP	ns	ns	
	ER-tag	***	ns	****
**Hs vs HEK**	GPI-GFP		ns	
	ER-tag	****	****	

Statistical significance at *** $p<0.001$, **** $p<0.0001$

Table S2. Statistical analysis (t-test) of the data presented in Figure 4.

MCF-7			
	24h	48h	72h
ER-tag vs N36Q	ns	ns	ns
ER-tag vs N52Q	*	ns	ns
ER-tag vs N36Q + N52Q	*	ns	ns
N36Q vs N52Q	*	ns	ns
N36Q vs N36Q + N52Q	*	ns	ns
N52Q vs N36Q + N52Q	ns	ns	ns
**MDA-MB-231**			
ER-tag vs N36Q	ns	ns	ns
ER-tag vs N52Q	*	****	****
ER-tag vs N36,52Q	ns	*	****
N36Q vs N52Q	ns	****	****
N36Q vs N36,52Q	ns	ns	****
N52Q vs N36,52Q	ns	ns	ns
**Hs578T**			
ER-tag vs N36Q	****	***	****
ER-tag vs N52Q	****	****	****
ER-tag vs N36,52Q	****	****	****
N36Q vs N52Q	ns	ns	*
N36Q vs N36,52Q	ns	ns	ns
N52Q vs N36,52Q	ns	ns	ns
**HEK293T**			
ER-tag vs N36Q	****	****	
ER-tag vs N52Q	****	****	
ER-tag vs N36,52Q	****	****	
N36Q vs N52Q	ns	****	
N36Q vs N36,52Q	*	****	
N52Q vs N36,52Q	ns	ns	

Statistical significance at *p<0.05, *** p<0.001, **** p<0.0001

Table S3. Statistical analysis (t-test) of data presented in Figure 5.

**Effect of kifunensine on Golgi localization**				
	Hs578T	MCF-7	MDA-MB-231	HEK293T
ER-tag	**	ns	*	**
N36Q	****	ns	****	ns
N52Q	*	ns	ns	ns
GFP-GPI	ns	ns	ns	ns
**Effect of kifunensine on ER-Golgi localization**				
ER-tag	ns	ns	****	****
N36Q	ns	ns	***	****
N52Q	****	ns	*	ns
GFP-GPI	ns	ns	ns	ns
**Effect of kifunensine on Golgi plus ER-Golgi localization**				
ER-tag	**	ns	***	****
N36Q	**	ns	*	****
N52Q	****	ns	**	ns
GFP-GPI	ns	ns	ns	ns

Statistical significance at *$p<0.05$, ** $p<0.01$, *** $p<0.001$, **** $p<0.0001$
